# Atypical Presentation of Retroperitoneal Fibrosis Causing Colonic Obstruction: A Case Report

**DOI:** 10.7759/cureus.55621

**Published:** 2024-03-06

**Authors:** Eleonora Achrak, Emily Manville, Mumen Ayyat, Ruben D Toribio

**Affiliations:** 1 Department of Surgery, Touro College of Osteopathic Medicine, New York, USA; 2 Surgery, Brookdale University Hospital and Medical Center, Brooklyn, USA

**Keywords:** fibroinflammatory disease, ormond’s disease, constipation, colonic obstruction, retroperitoneal fibrosis

## Abstract

Retroperitoneal fibrosis (RPF), also referred to as Ormond’s disease, is a rare fibroinflammatory condition characterized by abnormal fibrous tissue deposition in the retroperitoneal space, which traditionally presents with ureteral obstruction. Nonetheless, our case report showcases an exceptional instance involving a 70-year-old female patient who presented with symptoms suggestive of colonic obstruction, an unusual presentation that is not commonly associated with RPF. Although RPF has established associations with autoimmune conditions such as immunoglobulin G4-related disease and systemic lupus erythematosus, its connection to colonic obstruction remains undocumented in the medical literature.

Our patient is a 70-year-old female who presented with constipation, anemia, and fecal occult blood. Her past medical history included a hysterectomy due to fibroids, right breast lumpectomy, type 2 diabetes mellitus, subclinical hyperthyroidism, hypertension, and obesity. Upon physical examination, the patient’s abdomen appeared protuberant but was non-tender to palpation. Bowel sounds were normal, and there was no distension. Notably, there was no tenderness in the right or left costovertebral angles, nor was there any guarding. Workup with colonoscopy could not be completed due to the inability to pass a colonoscope beyond the rectosigmoid junction. Further workup with barium enema confirmed an apple core lesion seen in the rectosigmoid concerning for a neoplastic or inflammatory process. Finally, a computed tomography scan of the abdomen and pelvis showed a 7.1 cm right pelvic mass attached to the bladder and cecum, moderate right hydroureteronephrosis, and a 5.2 cm left adnexal mass with soft tissue changes narrowing the sigmoid colon. The next step was to take the patient for an exploratory laparotomy. During exploratory laparotomy, extensive adhesions and desmoplastic reactions were observed in the pelvic region, involving the sigmoid colon, bladder, cecum, and appendix. Two firm masses were identified in the retroperitoneum, one located in the left lower quadrant (LLQ) adherent to the posterior wall of the sigmoid colon and one in the right lower quadrant (RLQ) adherent to the posterior wall of the cecum. Three specimens were sent to pathology for further examination: a portion of the sigmoid colon, a resection from the RLQ mass, and a resection from the LLQ mass. Pathology reported dense fibrotic masses with abscess-like formation, reactive in nature and of unclear etiology, and negative for malignancy. They were negative for fibromatosis (β-catenin negative), and IgG4+/IgG+ was approximately 5%. Interestingly, the LLQ mass also contained remnants of the fallopian tube and ovary and benign cystic changes.

This case report presents a unique and atypical presentation of RPF, deviating from the conventional presentation of ureteral obstruction. The patient’s initial symptoms suggested colonic obstruction, a clinical scenario rarely linked to RPF. This case underscores the significance of considering diverse clinical presentations when diagnosing RPF, thereby expanding our comprehension of the condition’s clinical spectrum and ultimately refining patient care and management.

## Introduction

Retroperitoneal fibrosis (RPF) is a rare medical condition characterized by a chronic inflammatory and fibrotic process occurring in the retroperitoneal space, often leading to compression of nearby structures, particularly the ureters [1]. This compression can result in hydronephrosis and renal insufficiency [2]. RPF can manifest as either idiopathic or secondary to various factors, including medications, infections, malignancy, radiation therapy, trauma, or previous surgeries. Notably, idiopathic RPF has been linked to a systemic autoimmune disease, possibly triggered by an antigen called ceroid found in atherosclerotic plaques, and often features the presence of immunoglobulin G4 (IgG4) plasma cells [2,3].

Epidemiologically, RPF most commonly affects individuals between the ages of 40 and 60 years, with a significant male predominance, estimated at a male-to-female ratio of 2:1 or 3:1. The precise incidence of RPF remains uncertain but is estimated to range from 1 per 200,000 to 500,000 individuals per year [3]. The pathophysiology of idiopathic RPF is believed to involve a systemic autoimmune disease, and it is often associated with elevated levels of acute-phase reactants, autoantibodies, and other markers of autoimmune diseases [2]. Histologically, RPF exhibits a non-specific inflammatory process characterized by fibroblastic proliferation, hyalinized collagen, and infiltrates of macrophages, plasma cells, and lymphocytes. Notably, most plasma cells in RPF are positive for IgG4, suggesting a potential association with IgG4-related disease (IgG4-RD) [4-7].

Clinical presentation of RPF can include symptoms such as lower back or flank pain, testicular pain, nausea, generalized malaise, anorexia, weight loss, and hypertension [3]. To diagnose RPF, clinicians typically rely on imaging studies such as computed tomography (CT) scans and magnetic resonance imaging (MRI), along with laboratory tests assessing renal function and inflammatory markers, such as erythrocyte sedimentation rate (ESR) and C-reactive protein (CRP). Treatment strategies for RPF vary depending on clinical presentation. For example, urgent decompression with nephrostomy tubes or stents may be necessary for severe ureteral compression and hydronephrosis to protect renal function. Medical treatment often involves steroids and immunosuppressive agents to control inflammation [8]. Surgery is reserved for specific scenarios, including persistent mass encasement of important structures or when malignancy is suspected.

In current medical literature, the occurrence of bowel obstruction associated with RPF is quite uncommon. While there have been documented cases of small bowel obstruction linked to RPF, there is no record of colonic obstruction in association with this condition. One such case involved a patient who initially presented with a spontaneous perforation of the cecum [9]. Upon surgical repair of the cecum, RPF was discovered surrounding the kidneys and upper ureters. The patient later developed bilateral ureteral obstruction as well as small bowel obstruction due to RPF. In this case report, we aim to shed light on an exceptionally rare scenario: the occurrence of colonic obstruction in the context of RPF. While it may be rare, awareness of atypical presentations and complications is vital in providing comprehensive and effective patient care. Our case underscores the importance of further research into the etiology and presentation of RPF to enable more accurate and timely diagnoses of this condition.

## Case presentation

**Figure 1:**
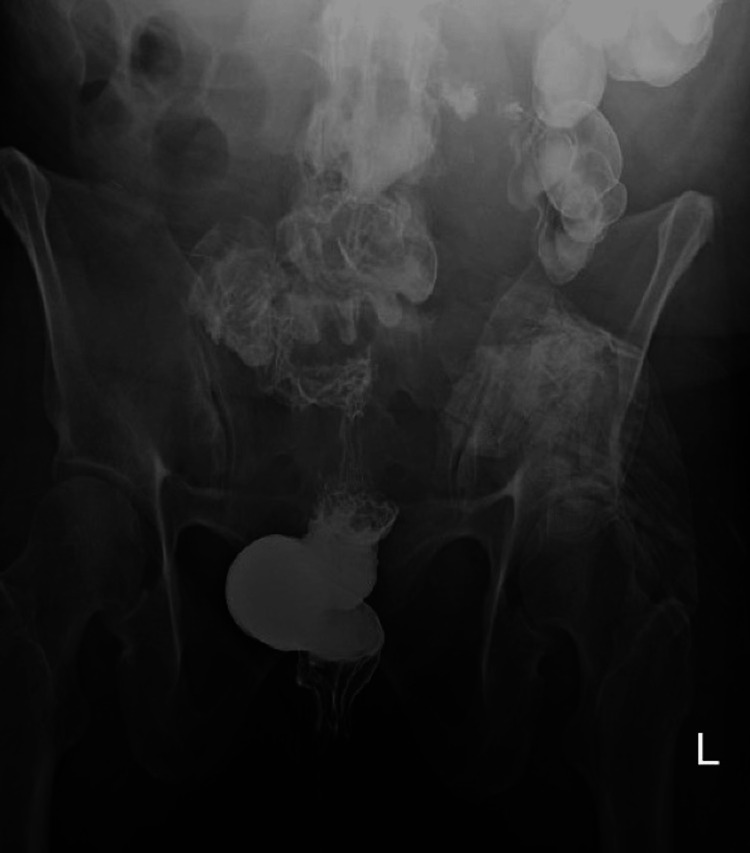
Barium enema demonstrates circumferential narrowing in the proximal and mid-sigmoid colon that has an “apple core” appearance.

We present the case of a 70-year-old female who presented in July 2023 with complaints of constipation for six months. She was found to be anemic and had fecal occult blood. Her past medical history included a hysterectomy due to fibroids, right breast lumpectomy, type 2 diabetes mellitus, subclinical hyperthyroidism, hypertension, osteoarthritis, and obesity. Upon physical examination, the patient’s abdomen appeared protuberant but was non-tender to palpation. Bowel sounds were normal, and there was no distension. Notably, there was no costovertebral angle (CVA) tenderness on the right or left, nor was there any guarding. Initial attempts at colonoscopy were thwarted by an inability to pass a scope beyond the rectosigmoid junction. A barium enema revealed an apple core lesion in the rectosigmoid, as shown in Figure 1.

**Figure 2:**
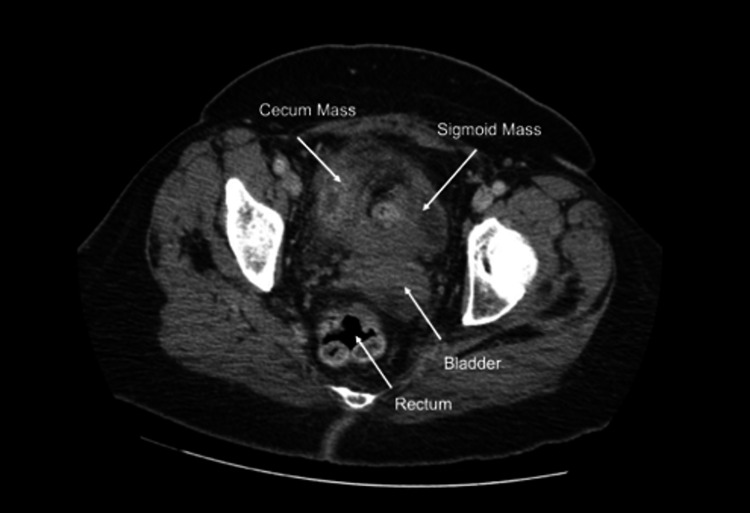
CT scan shows two discrete masses: one at the cecum and one at the sigmoid colon (transverse).

Subsequent abdominal and pelvic CT scans demonstrated two masses, one in the right lower quadrant (RLQ) and one in the left lower quadrant (LLQ) (Figure 2).

**Figure 3:**
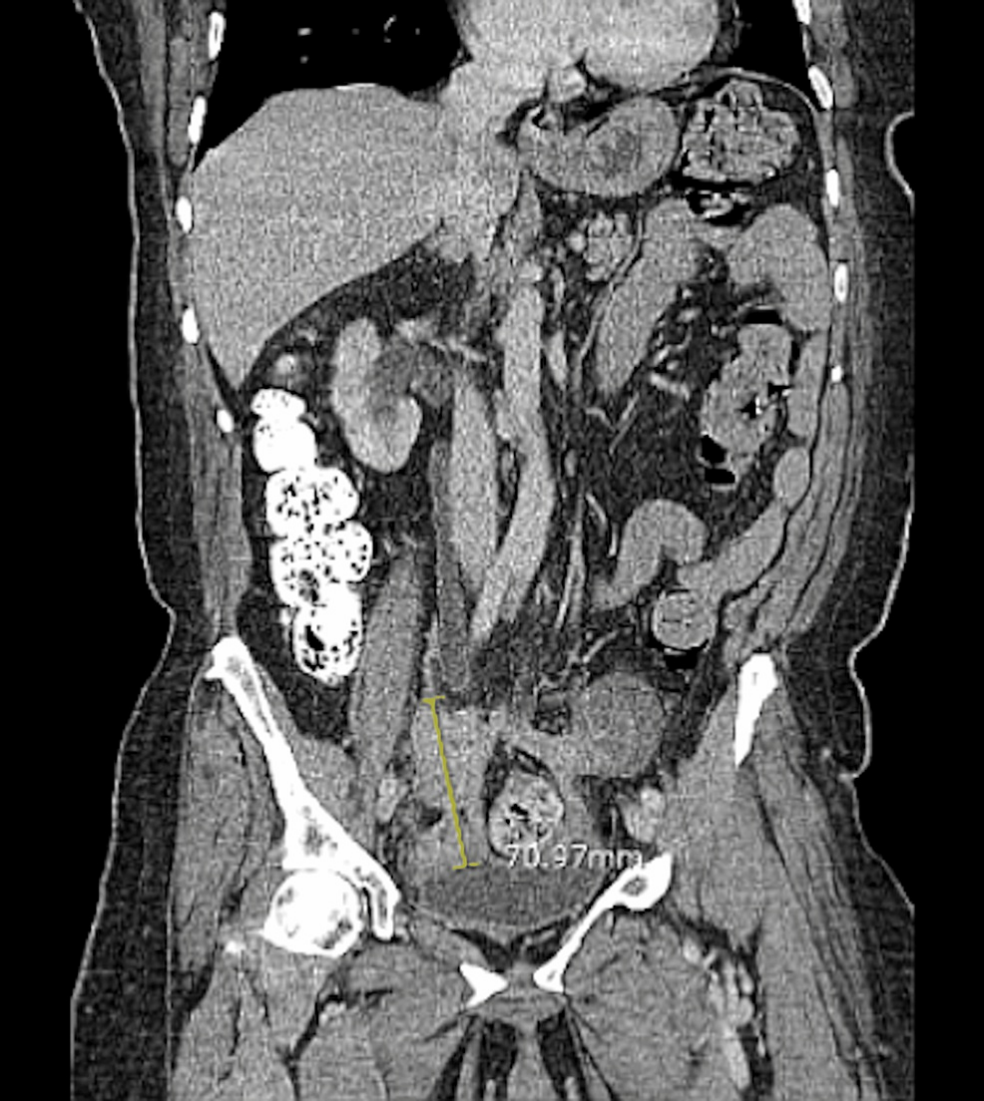
CT scan shows a cecum mass (coronal).

The RLQ mass measured 7.1 cm and was attached to the bladder and seemingly contiguous with the cecum (Figure 3).

**Figure 4:**
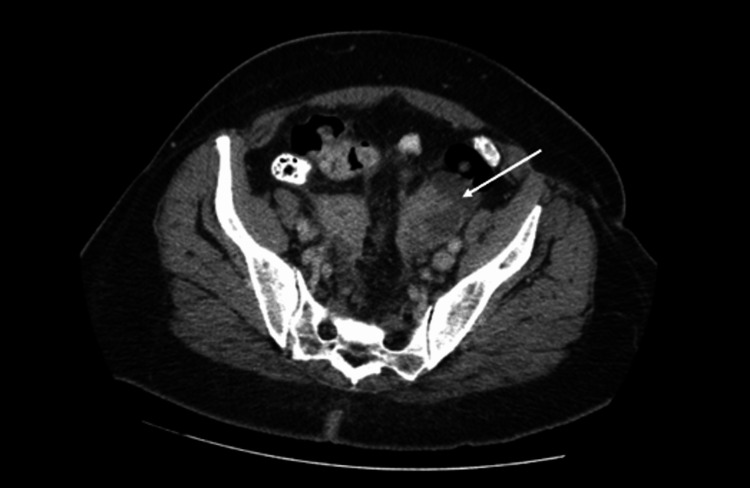
CT scan shows the stricture of the sigmoid colon by a soft tissue mass (transverse).

**Figure 5:**
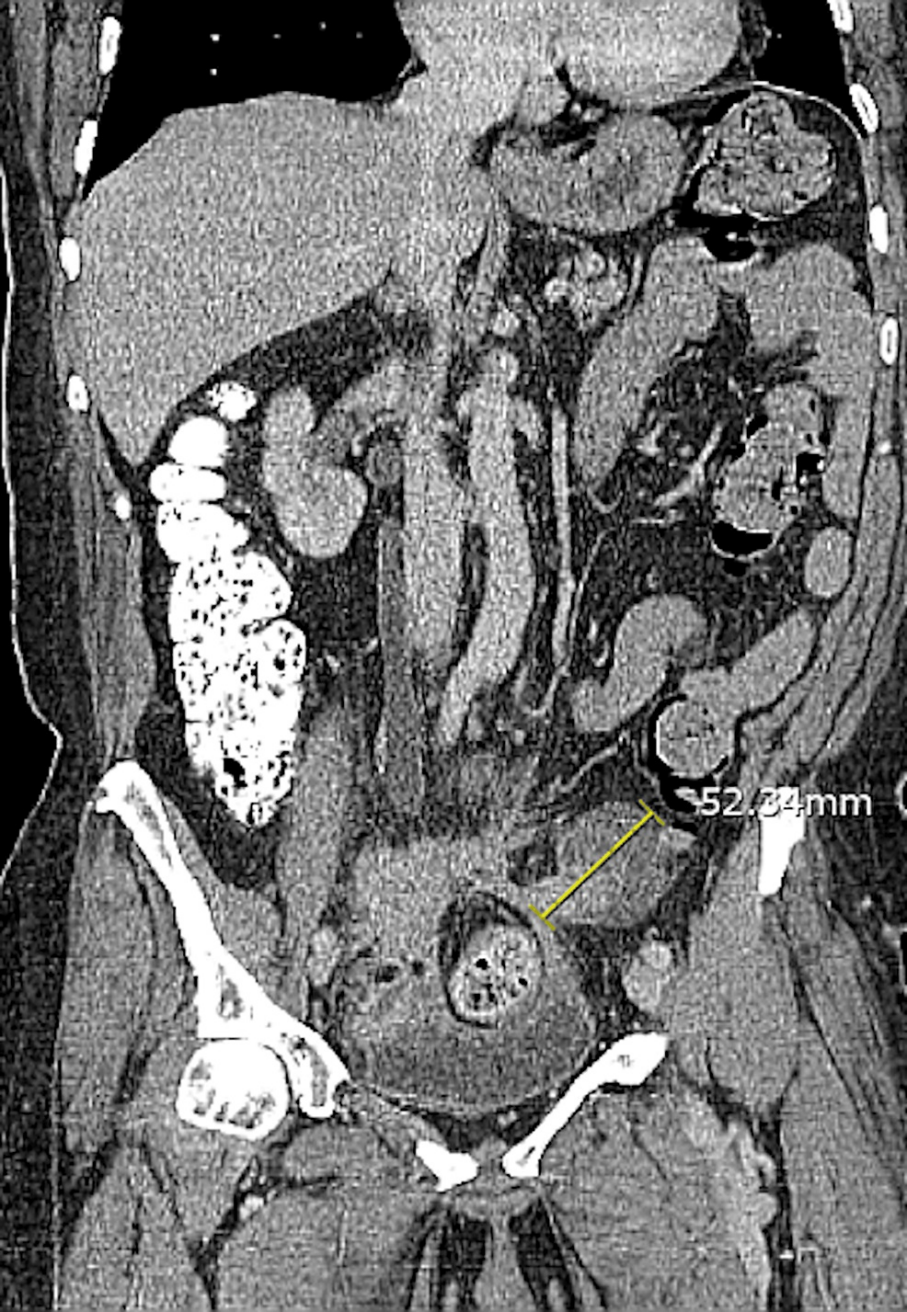
CT scan shows the stricture of the sigmoid colon by a soft tissue mass (coronal).

The LLQ mass measured 5.2 cm and had soft tissue changes narrowing the sigmoid colon (Figures 4, 5), positioned approximately 10 cm from the anal verge.

Additionally, moderate right hydroureteronephrosis to the level of the sigmoid colon was noted on the pelvic CT scan. The radiologist posited potential inflammatory, infectious, or neoplastic origins for these masses.

Subsequently, an exploratory laparotomy was performed and extensive adhesions were observed, primarily involving the omentum adhered to the abdominal wall below the umbilicus. Additionally, the pelvis exhibited extensive adhesions and a severe desmoplastic reaction affecting the sigmoid colon, bladder, cecum, and appendix. Confirming what was seen on the preoperative CT scans, two firm masses were identified in the retroperitoneum, one in the LLQ adherent to the posterior wall of the sigmoid colon and one in the RLQ adherent to the posterior wall of the cecum. A minimal amount of purulence was noted in the pelvis, and wound cultures were obtained. Lysis of adhesions and Hartmann’s procedure with mobilization of the splenic flexure were performed to remove the obstruction and a colostomy was created.

Three specimens were sent to pathology for further examination: a portion of the sigmoid colon, a resection from the RLQ mass, and a resection from the LLQ mass. Histopathological examination reported dense soft tissue fibrotic masses with abscess-like formation, reactive in nature and of unclear etiology, and negative for malignancy. They were negative for fibromatosis (β-catenin negative), and IgG4+/IgG+ was approximately 5%. Interestingly, the LLQ mass also contained remnants of the fallopian tube and ovary and benign cystic changes.

The patient had a postoperative course complicated by ischemia of the stoma and colostomy prolapse requiring stoma revision. She was discharged to a rehabilitation facility on the 24th postoperative day. She was seen at follow-up in the outpatient office one week later where she was ambulatory without complaints.

## Discussion

This case of RPF has many unique features that distinguish it from existing documented cases. Our patient’s presenting symptoms centered around colonic obstruction: constipation, anemia, and fecal occult blood. Importantly, she denied abdominal or flank pain or oliguria, and no CVA tenderness was elicited on physical examination. In one case series of patients with idiopathic RPF, 88% of patients complained of abdominal or back pain, with 83% of those patients having either bilateral or unilateral hydronephrosis on CT scans [10]. Furthermore, the CT scans of these cases demonstrated that the hydronephrosis was due to a periaortic focus of the RPF that surrounded the abdominal aorta and caused proximal obstruction of the ureter.

While the CT scan of our patient did show right-sided hydronephrosis, she had no symptoms related to the urinary obstruction. In addition, our patient’s hydronephrosis was due to obstruction of the distal right ureter. The distal location of the RPF is an important distinguishing factor in our case as it resulted in absent urinary symptoms and instead symptoms of bowel obstruction. While another case series showed that 13% of patients presented with constipation, these patients also endorsed abdominal or flank pain, with some also reporting testicular pain, varicocele, or hydrocele [10]. Our patient’s unique presentation of colonic obstruction-associated RPF distinguishes this case of RPF from what predominates in the literature.

The etiology of RPF is divided into idiopathic and secondary causes. Over 70% of RPF is classified as idiopathic [11]. It has long been proposed that idiopathic RPF is due to an autoimmune mechanism, and more recently postulated that it is related to IgG4-RD [5,11,12]. IgG4-RD is characterized by massive infiltration of lymphocytes and IgG4+ plasma cells in the tissue at a ratio of IgG4/IgG cells >40% [12]. In addition to retroperitoneal structures, IgG4-RD has also been documented in the lacrimal glands, salivary glands, lungs, pancreas, and kidneys [12]. Despite the association between idiopathic RPF and IgG4-RD, case studies like Zen et al. demonstrate that RPF can be classified as IgG4-RD related or IgG4-RD unrelated when looking at IgG4+/IgG+ ratio in affected tissue [4]. In our case study, our patient had a very low IgG4+/IgG+ ratio of 5% which is inconsistent with an IgG4-RD diagnosis of RPF. It has been posited that IgG+/IgG+ ratio >40% has a sensitivity of 94.4% and a specificity of 85.7% in the diagnosis of IgG4-RD [13]. Idiopathic RPF is frequently associated with autoimmune thyroiditis, systemic lupus erythematosus, rheumatoid arthritis, and psoriasis [14]. Not only is our case unrelated to IgG4-RD our patient did not have any diagnosis of any other autoimmune diseases. Our case’s atypical presentation of colonic obstruction and etiology unrelated to IgG4-RD or any autoimmune disease makes it exceedingly rare and important to further discussion and research on idiopathic RPF.

Our case also illustrates the challenges of conventional imaging for RPF. A CT scan can be used to reliably locate and evaluate the extent of RPF on the surrounding structures. Typically, the fibrosis is centered around the aortic bifurcation and may extend to the iliac arteries and ureters and further to the small intestine, pancreas, and spleen [15]. In our patient, however, the aorta was completely unaffected. Many have attested to the challenge of distinguishing benign RPF from neoplasia using CT scans. Some posit that malignant soft tissue masses tend to be bulkier and larger, causing a mass effect that displaces surrounding structures [15]. Others have tried to distinguish malignant and benign causes based on morphological features of lobularity and density [16]. While these approaches aim to simplify the distinction between benign and malignant causes, many cases have demonstrated the inability of these features to make accurate diagnoses [15-17]. In fact, in one such study, over one-third of cases with biopsy-proven RPF had no abnormalities on CT scans, namely, when the fibrosis was limited to the distal ureters and pelvis [17]. Our patient’s CT scan displayed features that were highly suggestive of a malignant mass, i.e., an irregular mass in the right pelvis invading the right superior bladder and another mass in the left adnexa showing circumferential soft tissue changes around the sigmoid colon resulting in extrinsic narrowing. These features noted on CT used in conjunction with the classic “apple core” narrowing of the sigmoid colon seen on barium enema taken with the symptoms of bowel obstruction seemed to strongly suggest malignancy. When compared to cases of carcinoma causing colonic obstruction, our case seemed to certainly emulate more malignant features than what is typically seen with RPF. This case demonstrates the challenge of reliably distinguishing RPF from malignancy using imaging modalities such as CT scans, especially when it presents atypically, as in our patient.

## Conclusions

This case demonstrates an atypical presentation of colonic obstruction due to RPF and supports the finding that idiopathic RPF may be unrelated to IgG4-RD. In addition, it alerts physicians that they must suspect RPF even in the absence of typical urinary obstruction symptoms or a periaortic soft tissue mass on a CT scan. Further research should be performed to distinguish the distinct etiologies of this rare disorder and elucidate better diagnostic criteria for RPF.
